# Iranian human genome project: Overview of a research process among Iranian ethnicities

**DOI:** 10.4103/0971-6866.60182

**Published:** 2009

**Authors:** Kambiz Banihashemi

**Affiliations:** Department of Medical Sciences, Great Persian Encyclopedia Foundation, Ministry of Science, Research and Technology, Tajrish, P.O. Box 19615-866, Tehran, Iran

**Keywords:** Genetic diversity, human genome project, Iranian ethnicities, population genetics

## Abstract

The Human Genome Project (HGP) refers to the international scientific research program, formally begun in October 1990 and completed in 2003, mainly designated to discover all the human genes, analyzing the structure of human DNA and determining the location of all human genes and also making them accessible for further biological and medical investigations. With the appropriate rationale approach, a similar study has been held in Iran. The study of human genome among Iranian ethnicities (IHGP) has been attempted formally in 2000 through a detailed and fully programmed research among all the major ethnic groups by more than 1,900 samples from all over Iran based on the main demographical and anthropological findings and formally known criteria considered for the international HGP. This paper overviewed the process of the research in the terms of program goals, primary data collection, research designation and methodology and also practical aspects and primary findings of the Iranian genome project and its progress during a nearly 5-year period.

## Introduction

The cells of everybody alive today, regardless of where or how they live, contain the same number of genes. Collectively known as “the human genome,” these genes contain all the information that makes us appear and function as humans rather than as members of some other species. Identifying each of the genes and locating its position on one of the human chromosomes was the first aim of the Human Genome Project (HGP), the on-going research project to which scientists from many countries have contributed. However, many human genes exist in more than one form (or “allele”) and we do not all carry exactly the same forms of every variable (“polymorphic”) gene. Awareness of the inheritance of both human appearance and behavioral characteristics and interfering with it dates from ancient times, which has appeared as a dream through some manipulations in other species and eventually implicated in the real humanitarian beings as the “*Human Genome Project*.”[1,2]

The HGP refers to the vast and great international effort, formally begun in October 1990 and completed in 2003, aimed at discovering all the estimated 20,000-25,000 human genes and make them accessible for further biological studies. There have been some other goals: To determine the complete sequence of the 3 billion DNA subunits and also as part of the HGP, parallel studies on selected model organisms such as the bacterium *E. coli* and the mouse to help develop the technology and interpret human gene function. At a glance, one may find that the project goals were to identify all the approximately 20,000-25,000 genes in human DNA, determine the sequences of the 3 billion base pairs in human DNA, store this information in databases and improve tools for data analysis, transfer related technologies to the private sector and, finally, address the ethical, legal and social issues that may arise from the project.

About 18 countries have participated in this worldwide effort, with significant contributions from the Sanger Center in the United Kingdom and research centers in Germany, France and Japan.[2,3,6,14]

## Iranian Human Genome Project

Iranian ethnicities are very different not only in their origins and languages but also in their cultures, life style and, obviously, their geographical distribution over the country. It is very important for the research purposes to identify these differences and classify these real differences to obtain a real framework on the major national plans of health care through the country. The main values of IHGP would be:

1. Enormous potentials for illuminating our understanding of Iranian ethnicities' history and identity.
2. The resource created by the IHGP will provide valuable information not only in the field of pure sciences but also in the form of genetic findings that interact with predisposition or resistance to endemic diseases.
3. The work of geneticists has been linked in an unprecedented way with that of anthropologists, archaeologists, biologists, linguists and historians, creating a unique bridge between science and the humanities in Iran for the first time.
4. Leading to greater understanding of the nature of differences between human populations, the Project had made a significant contribution to a closer understanding of Iranian ethnicities specifications.
5. Provide a better understanding of local and national differences between ethnicities and leads to better and more proportionate programs in the fields of the national health care system.

Similar extended patterns on the goals and aims of the analogue projects have been considered before in the literatures in international studies all over the world.[4-7,4,15,19]

### Leadership of the project

The main pivotal attempt in the project was establishing a scientific council. The council would be responsible for all scientific decision-making and would have a central observatory role in the process. Because they were deeply being tied to all research components and had to arrange them together, research centers did, in some sense, represent the administrative centerpiece of the genome project. However, centers' practices alone were not sufficient. Therefore, a major task force had been formed under the supervision of the council as a secretary department with all administrative duties to conduct and coordinate all the involved research centers around the country.[8,20]

Another important tool was establishing a variety of workshops and meetings designed to facilitate collaboration, assess the state of the art in a particular area and determine what actions are needed in every single step of the work. All the necessary ethical, legal and social considerations as international and domestic widely accepted values have been applied at every single step of the research process.[9,16]

### Iranian ethnicities

Using language as the major criterion, there are distinct human populations in Iran, whereas the cultural and religious groups in Iran added to this collection.[8,10,13,20] According to the findings of sociodemographical and anthropological studies in Iran, 10 major ethnic groups had been found at the time of the study to be associated with some conserved religious populations. The population combination in Iran at the time of sampling, in July 2001, was Fars 51%, Azari 24%, Gliaki and Mazandarani 8%, Kurds 7%, Arabs 3%, Lor 2%, Baloch and Zabolies 2%, Turkman 2% and others 1%.The last group consisted of the major religious and anthropologically unique population in the south of Iran. These populations were anthropologically unique or populations that constitute linguistic isolates and, finally, the populations that might be especially informative in identifying the genetic etiology of important disease.[11-14,5,7,8,11]

Samples from individuals within each of these populations were collected during a period of 3 months and the DNA content was analyzed to produce data on the frequency of occurrence within the population of an agreed set of alleles or other genetic markers. In order to be able to define the relationships between populations, a set of core markers had been studied in all the populations. The total numbers of samples per population on which these markers should be tested were needed to be large enough to ensure that the population could be characterized by a distribution of marker frequencies. The definition of a polymorphism, together with the number of samples that were feasible to consider handling for each population, had place constraints on the minimal frequency of variants that would be detected reliably in the samples collected for the HGP. Hence, wherever possible, samples should be as large as practical, and were at least 120 in each case.[4,5,10]

### Sampling strategies

The scientific council considered five possible sampling strategies to determine which one would be the most appropriate for a fruitful effort. Strategy III is the first of the three population-based sampling designs given in the following table. It records not only the geographic location of a sample but also the information provided about self-reported ethnicity, primary language, sex, age and parental birthplaces. Like the international committee on HGP, the Iranian scientific council on the IHGP believes that at a stage when genotyping technology is evolving rapidly, it would be scientifically inappropriate and premature to designate a common core set of markers that is to be genotyped in all samples. Thus, the level III strategy was selected as the main sampling strategy [Table 1].[8,20]

**Table 1:** Sampling strategies

	Non-population-based sampling		Population-based sampling		
	I Anonymous	II Location	III Identification data	IV Phenotypic data	V Pedigrees
Testable hypotheses	Genome evolution patterns of variation in the genome and overall genetic variation in humans	Same as I plus: Description and determination of spatial variation, such as variation of loci in space (migration)	Same as II plus: Patterns of migration, gene flow and population subdivision hypotheses from anthropology, archaeology, history and linguistics that should affect patterns of interpopulation variation. Preliminary studies on medically relevant loci and population-level medical associations	Same as III plus: Identify specific loci for possible biomedical applications, genotype interactions, within-group variation on medical and phenotypic data and associations between genes and phenotype at an individual level	Same as IV plus: Detailed studies on diseaseassociated genes

Accurate identification of population units for sampling purposes requires extensive knowledge of the social, political and linguistic composition of the region to be sampled and this stage of the study took a long time, as many as 2 years of collection of all preliminary necessary data. Published ethnographic studies can provide some of this knowledge and the most prominent anthropologists who work with the people have been consulted for the planning. If this information is not available, the secretary department should study the local situation in consultation with local leaders, experts and other researchers before designing the sampling strategy.[12,14,15,8,11]

In general, as many individuals as possible should be sampled in each population. However, for some phylogenetic purposes, 25 individual samples may be sufficient, provided that an adequate number of genetic markers are evaluated for each (e.g., markers from l00 to 200 different positions on the DNA).[19] In our study we at least have 120 samples. In addition to the biological samples of blood, a minimum set of sociodemographic data has been collected in a consistent manner from all individuals and populations. Whether in a regional center or in central repositories, serious consideration had been given to the criteria for storage because it is intended to preserve samples indefinitely.[3,20]

The blood samples were collected in two separate tubes individually, one containing EDTA for DNA extraction and the other containing ACD for cell-line preparation. A well-developed coding method has been applied to classify the samples according to their geographical and demographical specifications. In total, 1,982 samples were collected all over the aforementioned Iranian ethnicities through the time of this research.[8,16,17,20]

### The markers

Considering the clinical data on the endemic diseases and also based on the previous studies and regarding the international experiences, the markers had been classified as classical markers like RFLP markers, minisatellite markers, Y-chromosomal polymorphisms and microsatellite loci. Y-chromosome polymorphism has been searched in three separate sections in three departments and consists of the first group of reports in the line of data analysis, with the analyses of biallelic polymorphic markers as DYS 329, DYS 393, DYS 389 I and DYS 389 II. The second and third groups of Y-chromosome markers included the SRY465, 92R7 Tat, P12F2 and also Yap, 12F2, M9, M17 and M20, respectively. It should be noted that in this evaluation the Y-chromosome polymorphism had been considered by using small tandem repeats (STRs).

Among single-nucleotide polymorphisms (SNPs), the detection of common polymorphisms in GSTP1, P53, CYP2C9 and NQO1 had been the most important markers, which had been evaluated and considered for diseases like esophageal carcinomas among the Iranian ethnicities.

The diversity among Variable Number Tandem Repeats (VNTRs) had been searched in major loci as D1S80, D17S5, D19S20 and also APOB. And, at last, it is noteworthy that the mitochondrial haplogroups have been considered for their diversities among Iranian ethnicities. The last one would be important among these populations because of maternal origin tracking.

### Data collection and analysis

The direct product of the HGP initially had been genome maps and DNA sequences. In addition to database development, it was vital to deal with new findings and data analysis and interpretation of genome findings and DNA sequences. All the established and stored data had been processed with appropriate tools and some of them were converted to analyzed information. For maximum utility, by developing appropriate computer tools and information systems, the succeeding steps of the collection, storage and analysis of the immense amounts of data established a clear and standard set.[1,2,17]

## Findings and Future Perspectives

To know about the genetic diversity among humans, populations have been considered as a major guide point in the future research studies on the markers and will be the next steps conducted in scientific approaches toward diseases control and treatments. Some of the detailed results of the IHGP have been published up to now and the remaining will be published individually as a consequence of the project. The main points and global visions would be discussed here on a holistic approach to make a relatively thorough and more widened vision to the project.[8,20]

The Y-chromosome genomic complexity has been less than autosomal chromosomes with genetically useful markers in NRY and are thus more suitable for researches on the genetic history of mankind. One may consider the four microsatellites DYS392, DYS393, DYS389I and DYS389II more informative among all these markers. In Iran, the study of these mirosatellites has shown a differentiation in the frequency of expression but does not result in polymorphism for the DYS 393 while there is a significant polymorphic differentiation among these populations by considering the DYS389II, and this differentiation makes the marker a very good one for intrapopulation studies and legal medicine. The overall genetic variation among these populations has been belonging to the Fars ethnicity in which the highest haplotype diversity has been found.

The analyses of biallelic polymorphic markers such as 92R7 and SRY465, Tat and also P12F2 as a marker of the middle eastern population showed some differences among Iranian ethnicities. The mutation frequency of P12F2 for the Azari population was 4% while the frequency for the Fars and Kurd ethnicities was 0%. Also, the mutation frequency of Tat has been different among Azari and the other two aforementioned ethnicities, although neither this difference nor the abovementioned different frequencies has been proved to be statistically meaningful. The study of other Y-chromosome markers such as Yap also showed some differences among Azari ethnicity and the Kurd and Fars ethnicities and one may conclude that the Kurd and Fars ethnicities have been more consanguineous between themselves. The same theme has been true for M17, and the study showed a significant variation between the distributions of this marker among the Azari ethnicity, making a hypothetical link to Arian migration to Central Asia.

Among the other important markers, one may consider the SNPs and the more frequent of them in Iran, which lead to some common disorders. In IHGP, it has been defined that there are heterozygote alleles for P53 among the Mazandrani ethnicity, but the least among Kurds, something that has been established through northern Iranian susceptibilities to some cancers. Also, there have been genotypic similarities between Iranian and northern India tribes for markers like GSTP1, something that may give a clue on these population intermarriage and migrations through different periods of history.

The other important tracking markers in the IHGP were mitochondrial haplogroup diversity, and the study established some interrelation between the polymorphism in these haplogroups and genetically based serious disorders like LHON, multiple sclerosis and hypertrophic cardiomyopathies.

As the first steps of our understandings of the genetic diversity among people, these findings may help us obtain a more deepened and specific search for the local and regional health problems and endemic vulnerabilities to diseases that sometimes are so preventable and save the people from permanent disabling sequels.[18,19] Generally, all these segments of the study together gradually would complete the puzzle of our understandings of Iranian genome diversity and also the origins of the ethnicities and tribes. The main point in between would be a strong clue to the program on the major global or local health care and patient screening system. Certainly, when such a system focuses on the well-recognized goals and targets, it would be highly successful while maintaining the basic points of economical benefits. The more we know about the predispositions to disorders and its geographical, demographical and ethnicity-based distribution, the more accurate and beneficiary decision-making will happen.

On a global and much wider aspect, the results of such studies will help the world researchers and health care stakeholders to gain a broader and much clearer window toward the regional and continental problem-solving approaches and facilitate international coordination and cooperation in achieving the goal of a healthy world.
